# Safe Helicopter Landing on Unprepared Terrain Using Onboard Interferometric Radar

**DOI:** 10.3390/s20082422

**Published:** 2020-04-24

**Authors:** Pavel E. Shimkin, Alexander I. Baskakov, Aleksey A. Komarov, Min-Ho Ka

**Affiliations:** 1 Department of Radio Engineering Devices and Antenna Systems, Moscow Power Engineering Institute, National University, 111250 Moscow, Russia; shimkinpy@mpei.ru (P.E.S.); baskakovai@mpei.ru (A.I.B.); komarovaa@mpei.ru (A.A.K.); 2 School of Integrated Technology, Yonsei University, Seoul 21983, Korea

**Keywords:** interferometric radar, helicopter landing, simulation model

## Abstract

This letter proposes a radar interferometric survey system for the ground surface of helicopter landing sites. This system generates high-quality three-dimensional terrain surface topography data and estimates the slope of the site with the required accuracy. This study presents the processing algorithms of the radar system for safe helicopter landing using an interferometric method and also demonstrates the efficiency of the proposed approach based on computer simulation results. The results of the calculated potential accuracy characteristics of such a system are presented, as well as one of the variants of the algorithmic implementation of a simulation computer model implemented on MATLAB. Visual results of modeling using an example of a helicopter landing on a non-uniform surface relief similar to a real case are shown. The study focuses on the simulation of a unique on-board radar system, which allows helicopters to land on an unprepared site with a high degree of safety, having previously determined the presence of dangerous irregularities, inclines, foreign objects, and mechanisms on the site.

## 1. Introduction

One of the main causes of helicopter accidents [1,2] is the unreliability of means to ensure their landing on unprepared landing sites (LSs) in adverse weather conditions during the day and at night with poor visual visibility. Even in good weather conditions, owing to the dusty surface of the earth, the pilot and crew are at risk during landing. Massive dust clouds formed by air swirls owing to the helicopter’s screws substantially mask the LS. At the same time, irregularities with a height of 0.5 m and more and LSs with slopes more than 15° [2] already represent a danger to the landing of the helicopter, especially in strong winds. Existing on-board systems (satellite navigation systems, on-board radio altimeters) that most helicopters are equipped with cannot provide necessary information about the state of the terrain, slopes of the LS, and presence of foreign objects.

Until now, studies have been focused on two main areas of research in this field [3,4,5,6,7,8,9]. The first is the use of laser locators in the safe landing systems of a helicopter (SLSHs). High relief detailing is achieved and information about the LS relief is displayed on the screen in the cockpit. The main disadvantages of laser SLSHs are their strong dependence on weather conditions, i.e., it is impossible to survey the surface of the LS in the conditions of rain, fog, and snow, as well as their high cost compared to radar systems. The second is the use of radar systems in combination with special processing of signals reflected from the landing pad. Both continuous and pulsed systems with complex signals are used. There are several methods that allow information about the elevations of the surface relief to be isolated from radar data: stereoscopic, interferometric, clinometric, and polarimetric. Stereoscopic and interferometric methods require two images of the same surface area from different positions, the clinometric method works with only one image, and the polarimetric method requires a set of images taken with different signal polarizations.

Owing to a number of features of these methods, as well as flight regulation requirements [10], which discuss the need for mandatory flight of the proposed landing zone from several perspectives, a combination of the stereoscopic and interferometric methods is considered to be suitable for practical use when evaluating the surface topography.

The purpose of this work is to show the main stages of one of the options for the algorithmic implementation of a simulation model of the radar SLSH (RSLSH) interferometric method and also to demonstrate the performance of the proposed solution for safe landing of the helicopter based on the results of computer simulation.

## 2. Description of the RSLSH

**Figure 1:**
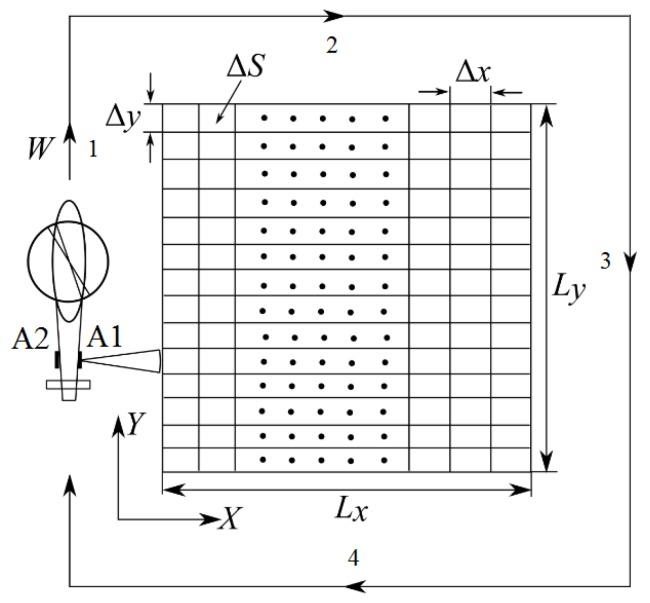
Imaging geometry of radar safe landing system of a helicopter (RSLSH).

To ensure a safe landing of the helicopter, a flight test is carried out when approaching it at a speed not exceeding 15 ms^−1^, according to flight regulations [10] from a height of approximately 50 to 100 m. During the flyby, a radar survey of the LS is carried out in the form of manual system. As the carrier moves, line-by-line scanning of the viewing area is performed using a narrow beam of a receiving-transmitting waveguide slot antenna in the azimuth plane without aperture synthesis; a wide beam in the slope plane is used to highlight the required area of view of the LS (Figure 1). It is important to note that during radar observation of the flight station, the helicopter must fly at a constant height with a constant speed.

An interferometer with a fixed base [11,12,13] in the form of a pair of antennae spatially separated by an interferometric base of a waveguide slot antenna mounted on a tail beam is used as a tool for measuring the relief of the LS and estimating the presence of foreign objects on the LS. One of the antennae works for reception and transmission, and the other only for reception. 

The operating frequency of the system is selected in the Ka-band, which is caused by minimizing the size of the antennae, ensuring high resolution of the on-board radar, as well as reducing the effect of losses on radio wave propagation.

High horizontal resolution $\Delta x=c/\left(2\Delta f\sin\theta_{1}\right)$ is provided by the use of a signal with a nanosecond duration, where Δf is the bandwidth of sensing signal; *c* is the speed of light; $\theta_{1}=\arccos\left(h/r_{1}\right)$ is the look angle; *h* is the flight altitude; and $r_{1}$ is the slant range.

The resolution in the azimuthal direction is determined by the size of the antenna, since at selected altitudes of the helicopter and the size of the LS, the radar operates in the azimuth plane in the near zone of the antennae.

A one-to-one relationship between the observation parameters and the interferometric phase difference (IPD) ϕ at the input of spatially separated receivers, which carries information about the resolution element, is determined by the relation [12,13]:

(1) $$z_{i}=h-r_{1}\cos\omega \sqrt{1-{\left(\left(r_{1}^{2}+b^{2}-{\left(r_{1}-\frac{\lambda }{4\pi }\phi \right)}^{2}\right)/\left(2r_{1}b\right)\right)}^{2}}-r_{1}\sin\alpha \cdot \left(\left(r_{1}^{2}+b^{2}-{\left(r_{1}-\frac{\lambda }{4\pi }\phi \right)}^{2}\right)/\left(2r_{1}b\right)\right).$$

where α is the inclination of the baseline from horizontal; λ is the wavelength; and *b* is the baseline. 

In accordance with Equation (1), the resolution of the resolution element is a function of many variables and theoretically, provided that the individual components are uncorrelated, the resulting error in estimating the relief of the LS is determined by the sum of the errors of each of the parameters included in Equation (1), e.g., Equation (2):

(2) $$\sigma_{z}^{2}=\sigma_{z\hat{\phi }}^{2}+\sigma_{zh}^{2}+\sigma_{zr_{1}}^{2}+\sigma_{zb}^{2}$$

where $\sigma_{z\hat{\phi }}^{2}$, $\sigma_{zh}^{2}$, $\sigma_{zr_{1}}^{2}$, and $\sigma_{zb}^{2}$ are the variance in the resolution element height due to the estimation error of the phase difference $\sigma_{\hat{\phi }}$, the measurement error of the altitude $\sigma_{h}$, the measurement error of the slant range $\sigma_{r_{1}}$, and the measurement error of the baseline length $\sigma_{b}$, respectively.

In order to determine the potential accuracy characteristic of the measurement of the relief of the LS with the help of the RSLSH, it is necessary to obtain a ratio only for the fluctuation error $\sigma_{z\hat{\phi }}$, since the remaining errors are inherently systematic and can be compensated for. The determining error of measuring the relief of the LS, as is known [12,13,14,15], is associated with the evaluation of the IPD $\hat{\phi }$ as seen in Equation (3):

(3) $$\sigma_{z\hat{\phi }}=\frac{\lambda h\tan\theta_{1}}{4\pi b\cos\left(\theta_{1}-\alpha \right)}\sigma_{\hat{\phi }};\;\sigma_{\hat{\phi }}=\frac{1}{\sqrt{2N}}\frac{\sqrt{1-\gamma^{2}}}{\gamma }$$

where $\sigma_{\hat{\phi }}$ is the root mean square (RMS) error of IPD estimate; *N* is the number of incoherent integration; and γ is the correlation coefficient for two received signals in the interferometer.

The used interferometer with a fixed baseline is characterized by the decorrelation of paired echoes coming to the spatially separated antennae of two receivers $\gamma_{spatial}$ and due to thermal noise in system $\gamma_{noise}$.

For each of the factors, analytical expressions are derived and the resulting correlation coefficient is determined by using Equation (4), under the assumption that the real surface is a distributed radar target consisting of a set of independent partial reflectors inside the resolution element whose applets are distributed according to the normal law [12]:

(4) $$\begin{array}{c} \gamma =\gamma_{spatial}\cdot \gamma_{noise};\;\gamma_{noise}=\frac{1}{1+snr^{-1}};\\ \gamma_{spatial}=\left(1-\frac{2b\cos\left(\theta_{1}-\alpha \right)}{\lambda r_{1}\tan\theta_{1}}\Delta r\right)\cdot \exp\left[-2\pi^{2}{\left(\frac{\sigma_{h}b\cos\left(\theta_{1}-\alpha \right)}{\lambda r_{1}\sin\theta_{1}}\right)}^{2}\right] \end{array}$$

where Δr is the slant range resolution; $\sigma_{h}$ is the RMS of small irregularities on the surface of a large relief; and *snr* is the single-to-noise ratio.

The final expressions for the standard deviation of the estimate of the applicability of the relief through the standard deviation of the estimates of the IPD are obtained by substituting Equation (4) into Equation (3).

**Figure 2:**
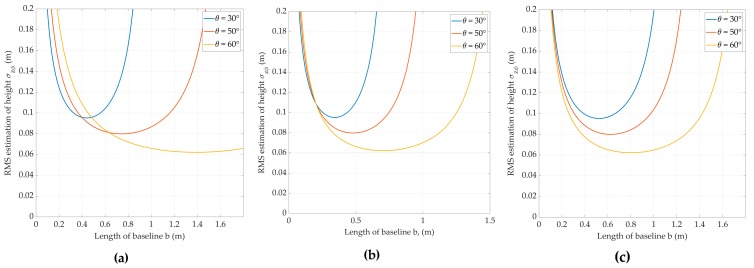
Height estimation error due phase estimation error on the baseline length at different look angles: (**a**) α=0°; (**b**) α=45°; (**c**) α=90°.

As a result, with the parameters of the RSLSH: $f_{c}=35$ GHz, *h =* 75 m, $\theta_{1}=30{}^{\circ}\sim 60{}^{\circ}$, *N* = 4, Δr=0.5 m, Δy=0.8 m, $\sigma_{h}=7,77\cdot 10^{-3}$ m, and *snr* = 13 dB, we have the following dependence of the standard deviation of the relief estimate on the size of the interferometer base at different look angles (Figure 2).

According to Figure 2, it is preferable to choose the size of the fixed baseline of the interferometer to be from 0.48 to 0.57 m, at which the potential values of the accuracy of the measurements of the LS surface will be in the range of approximately from 6 to 10 cm.

## 3. Numerical Simulation

### 3.1. Structure of the Simulation Model

The software package MATLAB is used as the simulation platform; the primary toolset for radar simulation with this software is the Phased Array System Toolbox, as in [16]. The simulation process can be divided into the following stages: (1) setting the Digital Elevation Model (DEM) and its parameters; (2) setting the parameters of the interferometric system;(3) simulation of the trajectory signal, its processing, and synthesizing the radar images; (4) calculation of the IPD; (5) interferometric processing to obtain an elevation map as the final output.

### 3.2. Digital Elevation Model

**Figure 3:**
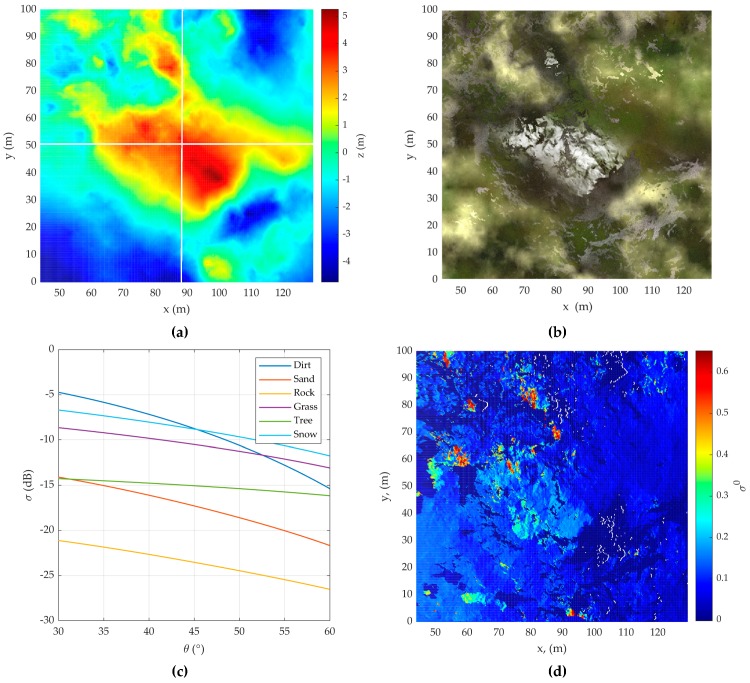
(**a**) Digital elevation model (DEM) for acquisition 1; (**b**) optical image of DEM for acquisition 1; (**c**) radar cross section (RCS) for some types of surface; (**d**) RCS of DEM for acquisition 1.

At this stage, the terrain features are generated according to the phenomenological surface model [11,12]. Each resolution element on the Earth’s surface is represented by a set of normally distributed partial scatterers, on which scattering conditions known from the experimental results are imposed. Illustrative simulation results are shown on the example of a user-defined DEM shown in Figure 3a. By type, the surface consists of water, sand, soil, grass, and snow, the optical image of which is shown in Figure 3b.

As a model of the radar cross section (RCS) for surfaces such as grass, trees and snow, an experimentally obtained full-scale model RCS for various types of surfaces is used, which is valid for the microwave frequency range from 3 to 95 GHz. It takes into account the standard deviation of fine surface roughness $\sigma_{h}$, the look angle on the surface θ, and the wavelength λ, and has the following form [17] (Equation (8)):

(5) $$\sigma^{0}\left(\theta ,\sigma_{h},\lambda \right)=A{\left(\frac{\pi }{2}-\theta +C\right)}^{B}\exp\left[-D/\left(1+\frac{0.1\sigma_{h}}{\lambda }\right)\right]$$

where A, B, C, and D are empirical model coefficients. In [14], the values for these constants are given in the frequency range from 3 to 95 GHz for the indicated types of surfaces.

The RCS model for surfaces such as soil, sand, and stone is a semi-empirical model of backscattering of the earth’s surface [18,19,20] for three types of polarization. For them, backscatters from the four surfaces are simulated using the semi-empirical model for the backscattering coefficient $\sigma^{0}$ in three polarizations: horizontal (HH), vertical (VV), and cross-polarization (HV) [18]:

(6) $$\sigma_{VV}^{0}=g\frac{\cos^{x}\theta }{\sqrt{p}}\left[\Gamma_{VV}\left(\theta \right)+\Gamma_{HH}\left(\theta \right)\right],\;\sigma_{HH}^{0}=p\sigma_{VV}^{0},\;\sigma_{HV}^{0}=q\sigma_{VV}^{0}$$

where $p={\left[1-{\left(\frac{2\theta }{\pi }\right)}^{\frac{1}{3\Gamma_{0}}}\exp\left(-0,4k\sigma_{h}\right)\right]}^{2}$; $g=2,2\left[1-\exp\left(-0,2k\sigma_{h}\right)\right]$; $\Gamma_{0}={\left|\frac{1-\sqrt{\varepsilon_{r}}}{1+\sqrt{\varepsilon_{r}}}\right|}^{2}$ is the reflection coefficient for normal incidence; $\Gamma_{VV}\left(\theta \right)$ and $\Gamma_{HH}\left(\theta \right)$ are Fresnel’s reflection coefficients for oblique incidence at angle θ; $\varepsilon_{r}$ is the relative permittivity $q=0,23\Gamma_{0}^{0,5}\left[1-\exp\left(-0,5k\sigma_{h}\sin\theta \right)\right]$; and $x=3,5+\frac{1}{\pi }\tan^{-1}\left[10\left(1,64-k\sigma_{h}\right)\right]$.

### 3.3. Synthesis of Radar Images and IPD Processing

If we denote the radar images obtained during two intervals or sub-intervals of observations as ${\dot{P}}_{1}$ and ${\dot{P}}_{2}$, we can then obtain an interferogram from their pixel-by-pixel complex conjugate multiplication using Equation (7):

(7) $$I_{P_{1}P_{2}}\left(x,y\right)={\dot{P}}_{1}\left(x,y\right)P_{2}^{*}\left(x,y\right)=\left|P_{1}\left(x,y\right)\right|\cdot \left|P_{2}\left(x,y\right)\right|\exp\left\{j\left[\phi_{P_{1}}\left(x,y\right)-\phi_{P_{2}}\left(x,y\right)\right]\right\}$$

and the interferometric phase difference can be defined as the argument of the multiplication result as seen in Equation (8):

(8) $$\phi_{P_{1}P_{2}}\left(x,y\right)=\arg\left\{\sum_{n=1}^{N}I_{P_{1}P_{2}}\left(x,y\right)\right\}=\sum_{n=1}^{N}\left[\phi_{P_{1}}\left(x,y\right)-\phi_{P_{2}}\left(x,y\right)\right]$$

**Figure 4:**
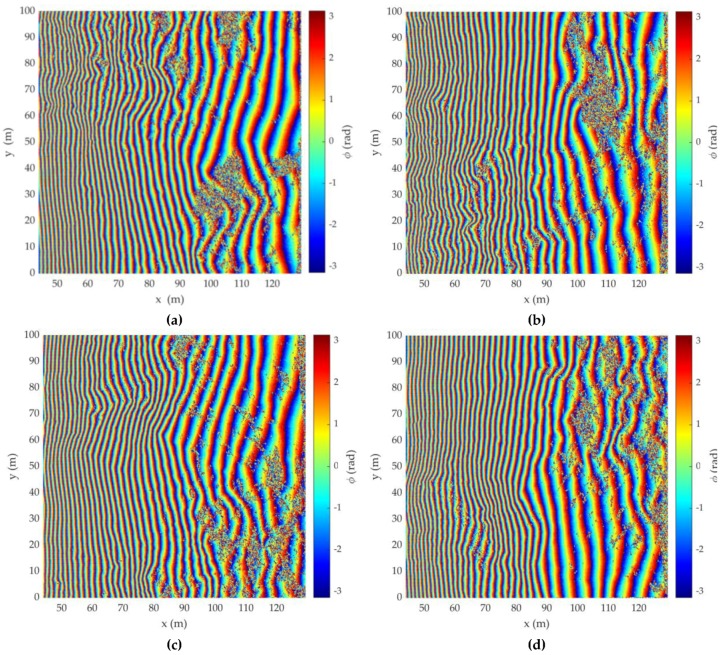
Interferometric phase difference (IPD) of DEM of (**a**) acquisition 1; (**b**) acquisition 2; (**c**) acquisition 3; (**d**) acquisition 4.

Figure 4 illustrates the interferometric phase difference (IPD) for DEMs, which are rotations by 90, 180, and 270° anticlockwise from the acquisition 1 simulation model, respectively. 

**Figure 5:**
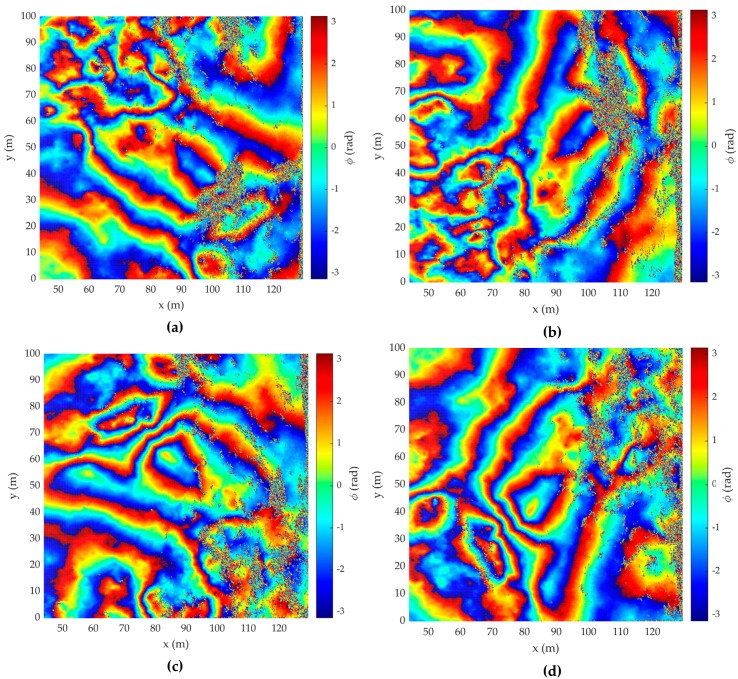
IPD after removing the flat Earth IPD of (**a**) acquisition 1; (**b**) acquisition 2; (**c**) acquisition 3; (**d**) acquisition 4.

The standard interferometric processing followed that described in References [16,17,21,22,23] and included: elimination of the linear phase component along the range by subtracting the phase of the flat Earth from the IPD of the DEM; removing to the effects of the flat surface of the Earth (Figure 5); and elimination of the phase ambiguity.

**Figure 6:**
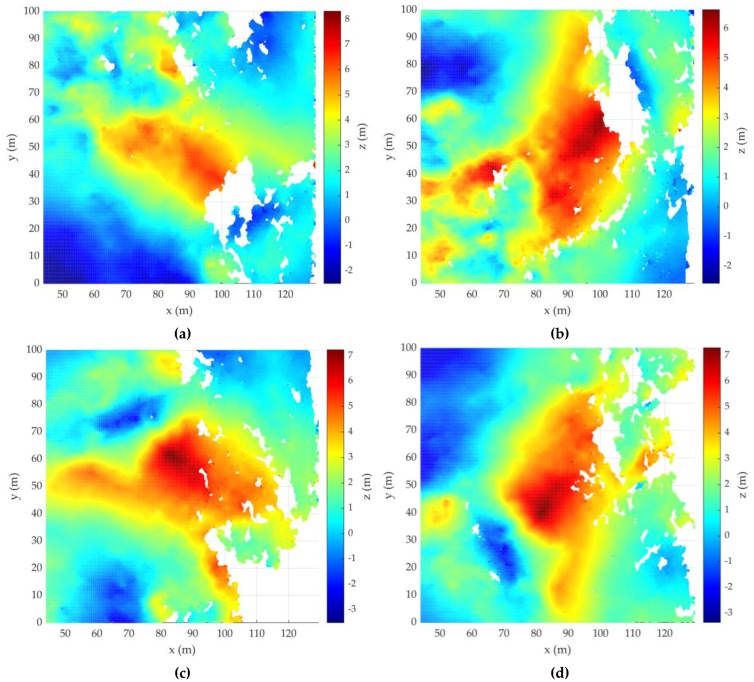
Estimation of DEM of (**a**) acquisition 1; (**b**) acquisition 2; (**c**) acquisition 3; (**d**) acquisition 4.

As the IPD may significantly exceed two during elevation changes, the recovery of the true phase difference from the IPD reduces to the interval (−π, π] and must be processed in an approach known as phase unwrapping (Goldshtein et al. (1988)). The scaling of the unwrapped IPD and generation of the DEM according to the unambiguous relationship between terrain elevation and change of IPD is shown in Figure 6.

**Figure 7:**
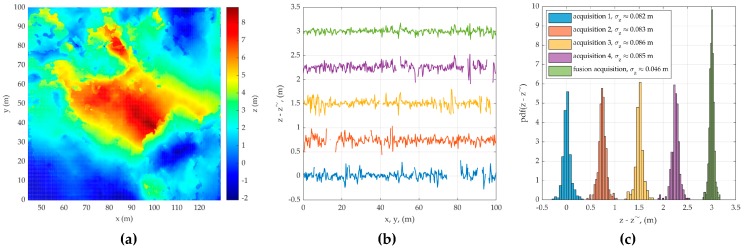
(**a**) combined DEM; (**b**) standard deviation of the errors; and (**c**) its histograms.

Taking into account only the phase component, the error in estimating the topography of the surface and its histogram in the selected sections (white vertical and horizontal lines in Figure 3a) for four observations are added in Figure 7a. Here, in order to prevent the graphs from merging into one, the value errors added a constant component multiple of 0.75 m depending on the observation number. The standard deviations of the estimation errors are in the range from 0.082 to 0.086 m, which is consistent with the theoretically calculated value.

## 4. Conclusions

In this article, we analyzed the algorithm of a proposed radar interferometric remote sensing system for a helicopter LS surface using an airborne radar. This algorithm helps in obtaining a high-quality radar image of the LS, which shows surface variation characteristics with sufficient accuracy to confidently determine the type of LS and the presence of unknown objects on it, by using a linear 3D model of the surface.

First, after illuminating the LS surface using electromagnetic waves, we obtained radio contrast patterns according to the backscattering from the resolution elements. Then, the resulting contrast pattern was superimposed with the phase difference information, covering the resolution elements of LS. This was the starting point of reconstructing the LS terrain topography. In this approach, the visualization of man-made objects on the LS is significantly improved. Our work shows that the measurement accuracy of the variations in the z coordinate was most significantly affected by the error in the measured phase differences of the interferometer signals. Therefore, the detection probability increased with an increase in the number of measurements.

Therefore, the proposed method demonstrates the ability to significantly improve the visualization of man-made objects at a helicopter LS using the phase difference information of the reflected signals reaching both antennae. However, the detection of sharp variations in LS terrain, such as hills and ravines, must be performed by considering the background-to-noise ratio. Phase-difference information helps to highlight large surface roughness in radar images and determine their relative heights.

According to the results of this research, the proposed algorithm can be applied for the safe landing of a helicopter under conditions of insufficient a priori information on the LS. According to flight regulations, helicopters fly around an expected landing site to determine the topography, slopes, and presence of unknown objects; then, the pilot makes a decision about landing.

The results can be a theoretical and implementation basis for the safe landing of a helicopter for building perspective onboard radar systems, choosing the geometry of LS illumination, and calculating the optimal performance of the system. This can detect the roughness and disturbing objects on the LS and increase the reliability of a safe landing in a dusty environment under day and night conditions, as well as under harsh weather conditions. 

In this work, the algorithm of the radar interferometric recording of the surface for the on-board radar was simulated, which made it possible to obtain a high-quality 3D image of the relief with the definition of the nature of the relief with the required accuracy.

The results of the simulation of interferometric signal processing RSLSH confirmed the possibility of its use as a promising tool in determining hazardous irregularities and foreign objects at the landing site from the resulting differential-phase interferometric images from the helicopter.

The main advantage of using RSLSH compared with other methods of safe landing of a helicopter on an unprepared site is that it is independent of the weather conditions and time of day.
